# Femtosecond coherence dynamics of exciton–polaritons

**DOI:** 10.1093/nsr/nwaf493

**Published:** 2025-11-19

**Authors:** Haoyuan Jia, Junhui Cao, Fei Chen, Fangying Peng, Yihui Li, Yihan Xu, Leizhu Chen, Ziyu Ye, Xianyan Zhao, Shian Zhang, Jietai Jing, Hongxing Xu, Zhanghai Chen, Tim Byrnes, Hui Li, Alexey Kavokin, Jian Wu

**Affiliations:** State Key Laboratory of Precision Spectroscopy, East China Normal University, Shanghai 200241, China; Abrikosov Center for Theoretical Physics, Moscow Center for Advanced Studies, Moscow 141701, Russia; Peking University Yangtze Delta Institute of Optoelectronics, Nantong 226010, China; State Key Laboratory of Precision Spectroscopy, East China Normal University, Shanghai 200241, China; State Key Laboratory of Precision Spectroscopy, East China Normal University, Shanghai 200241, China; State Key Laboratory of Precision Spectroscopy, East China Normal University, Shanghai 200241, China; State Key Laboratory of Precision Spectroscopy, East China Normal University, Shanghai 200241, China; State Key Laboratory of Precision Spectroscopy, East China Normal University, Shanghai 200241, China; State Key Laboratory of Precision Spectroscopy, East China Normal University, Shanghai 200241, China; State Key Laboratory of Precision Spectroscopy, East China Normal University, Shanghai 200241, China; State Key Laboratory of Precision Spectroscopy, East China Normal University, Shanghai 200241, China; State Key Laboratory of Precision Spectroscopy, East China Normal University, Shanghai 200241, China; Department of Physics, College of Physical Science and Technology, Xiamen University, Xiamen 361005, China; State Key Laboratory of Precision Spectroscopy, East China Normal University, Shanghai 200241, China; NYU-ECNU Institute of Physics, New York University Shanghai, Shanghai 200124, China; Center for Quantum and Topological Systems (CQTS), NYUAD Research Institute, New York University Abu Dhabi, Abu Dhabi, UAE; Department of Physics, New York University, New York 10003, USA; State Key Laboratory of Precision Spectroscopy, East China Normal University, Shanghai 200241, China; Chongqing Key Laboratory of Precision Optics, Chongqing Institute of East China Normal University, Chongqing 401120, China; Abrikosov Center for Theoretical Physics, Moscow Center for Advanced Studies, Moscow 141701, Russia; School of Science, Westlake University, Hangzhou 310030, China; Russian Quantum Center, Skolkovo Innovation Center, Moscow 121205, Russia; State Key Laboratory of Precision Spectroscopy, East China Normal University, Shanghai 200241, China; Chongqing Key Laboratory of Precision Optics, Chongqing Institute of East China Normal University, Chongqing 401120, China; Collaborative Innovation Center of Extreme Optics, Shanxi University, Taiyuan 030006, China

**Keywords:** optical-driven coherence transfer, exciton–polaritons, ultrafast dynamics

## Abstract

Exciton–polaritons perform as ideal carriers of macroscopic quantum coherence, which can be potentially manipulated through precisely shaping the driving laser field. However, the connection of the coherence properties between the pumping laser and the strongly coupled light-and-matter system is studied to a lesser extent. In this paper, we visualize the femtosecond dynamics of coherence transfer from the driving laser field to the resonantly excited exciton–polariton by an interferometric measurement. The resonant polaritons can effectively preserve the coherence of the pumping laser field in femtosecond timescales. At a high excitation strength, non-resonant polaritons appear at higher energies delayed by several picoseconds, without the phase coherence from the pump, which is understood by a coupled oscillator model. Our results offer the possibility of regulating the polariton coherence by finely shaping the external pump laser fields.

## INTRODUCTION

Exciton–polaritons (EPs), quasiparticles resulting from the strong coupling between cavity photons and electron-hole pairs, can form macroscopic quantum states through non-equilibrium Bose–Einstein Condensation (BEC) [1–4]. Polariton BEC has been realized at high temperatures and even at room temperature in various microcavity systems benefiting from the extremely light effective masses inherited from the photonic component [5–7]. It is thus an ideal platform for the study of macroscopic coherence [8]. Polaritons can condense, accompanied by spontaneous coherence buildup in the part-light part-matter EPs relaxing from an exciton reservoir. To understand the processes governing this, it is important to know if the initial coherence of the external driving field can be preserved during condensation formation or the non-linear scattering process. The spatial and temporal coherence measurement of EP systems have been investigated [9–12], showing a distinct growth-and-decay feature of first-order coherence function g^(1)^ and second-order coherence function g^(2)^, when the driving strength goes beyond the condensation threshold [13,14]. It is estimated that polaritons produced by resonant excitation can preserve the coherence of the pumping laser, while those produced by non-resonant excitation cannot due to the complex intermediate processes [15,16]. However, a clear observation on the coherence transfer from the pumping laser field to the polaritons under resonant excitation is yet to be demonstrated.

The major difficulty in investigating the dynamics of coherence transfer with resonant excitation comes from the fact that the resonantly induced EPs form at femtosecond timescales and the polariton signal inevitably overlaps with the pumping laser in the energy domain. In this work, by combining the femtosecond angle-resolved spectroscopic imaging (FARSI) technique [17,18] with precision temporal and frequency control of the femtosecond pumping laser pulses, we visualize the ultrafast coherence transfer dynamics from the pumping laser to the EPs in a ZnO whispering gallery mode (WGM) microcavity at room temperature. Under resonant excitation, EPs at energies resonant to the pump energy form, as well as at non-resonant higher energy levels. We investigate the underlying connection between the resonant and non-resonant polaritons. Based on an interference measurement of the pump laser and the photons originating from the EPs, unambiguous evidence of the coherence transfer from the pump laser to the resonantly excited EPs is characterized by measuring the first-order coherence function g^(1)^(τ). It is shown that the coherence is quickly lost in the later-emerging non-resonant polaritons. We consider that Rabi oscillation is the mechanism that populates the exciton reservoir and results in the subsequent formation of the non-resonant polaritons. Our work reveals the excitation pathways and mechanism of coherence transfer from the pump field to EPs, opening an avenue for coherent manipulation of macroscopic quantum states by precisely shaping the driving laser field.

## RESULTS AND DISCUSSION

### Driving laser field

Femtosecond pump laser pulses with a full width at half maximum (FWHM) energy of ∼6 meV are produced from a Ti:Sapphire laser system through a 4-f pulse modulator. A single lower polariton (LP) branch of the ZnO microcavity can be resonantly injected and EPs can be produced in the same energy region. A replica of the broadband femtosecond laser pulse (which we refer to as the reference laser pulse), generated by bypassing the 4-f pulse modulator, is precisely controlled by a delay stage and is utilized for the interference measurement. The energy FWHM of the reference laser pulses is ∼49 meV, which covers a wider energy range for further interference measurements (see Fig. S1). The pulse duration of the pump and the reference laser pulses are ∼400 and ∼200 fs (the difference originates from the spectral shaping), respectively. The pump laser is normally incident on the ZnO microcavity, and EPs formed at different LP branches can be explicitly measured through photoluminescence (PL) detection.

### Femtosecond coherence dynamics at low pump fluence

**Figure 1.**
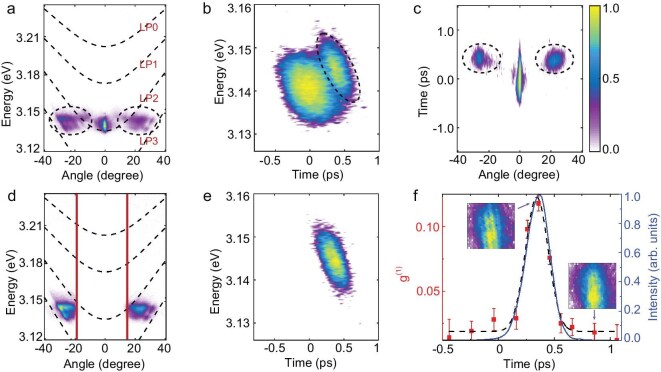
Resonant polariton excitation at low pump fluence. (a) Time-integrated angle-resolved spectra obtained at the pump fluence of ∼2.7 mJ/cm^2^. The energy is matched with the LP2 branch pumped resonantly at the ground state. The black dashed parabolas represent the fitted WGM dispersion curves of ZnO microcavity. (b and c) The energy- and angle-resolved dynamics of the pump laser and the PL from the resonantly excited EPs. Black dashed circles indicate the polariton PL emission. (d) The time-integrated angle-resolved spectra with the same conditions as (a) with a block for the pump laser. The area between the two red lines is the blocked region. (e) The corresponding dynamics in the energy domain. (f) Time-resolved coherence obtained between the reference laser and the EP signal compared with the EP signal intensity. The red datapoints are the extracted g^(1)^ (*τ*), which is fitted by a Gaussian function (black dashed line). The solid blue line is the experimentally obtained EP signal intensity. The insets are the measured interference patterns in real space after the Mach–Zehnder interferometer at time delays of ∼0 fs (left) and ∼700 fs (right).

Figure 1a shows the typical time-integrated angle-resolved spectrum of the PL emission at a pump fluence of ∼2.7 mJ/cm^2^, where the LP2 was pumped resonantly. The angle here represents the in-plane momentum, *k*_//_. The bright spot at around *k*_//_ = 0 mainly originates from the resonantly excited polaritons and the pump laser. EPs can be resonantly produced at this specific energy and momentum, which are quickly scattered through polaritonic interparticle interactions towards both sides in the momentum space and emerging at angles from 10° to 30°, labeled by the black dashed circles. The ultrafast dynamics in the angle and energy domain can be explicitly observed with femtosecond time-resolved measurements based on the FARSI technique, as shown in Fig. 1b and c. The black dashed circles indicate the EP signals, well distinguished from the pumping laser. A scattering time (defined as the time difference between the source and the emergent scattered signal) of ∼400 fs can be extracted for the EPs at ∼25° with respect to the source at 0° with a red shift of ∼10 meV in energy. The resonant polaritons appear and vanish almost simultaneously with the pump laser due to the limited external supply. The lifetime of these resonant polaritons (∼210 fs) is much shorter than the lifetime of those generated by non-resonant excitation, which are fed by the reservoir formed by excitons [19].

By placing a tiny block at the small angular region close to 0°, the signal from the pump laser can be eliminated (as shown in Fig. 1d and e), thus leaving the resonantly populated EPs for further interference measurement. The PL from the EPs and the temporally controlled reference laser are then sent into a Mach–Zehnder interferometer. Their relative time delay is tuned and the interference fringes in space are recorded by a charge-coupled device (CCD) detector. As shown in the inset in Fig. 1f, clear fringes show up close to zero time delay when the resonant EPs appear, but the fringes vanish at longer time delays. The g^(1)^ (*τ*) was extracted as the visibility of the interference fringes to describe the dynamics of coherence transfer, defined as (*I*_max_ − *I*_min_)/(*I*_max_ + *I*_min_). Here, *τ* represents the time delay between the reference laser pulse and the PL signal, and *I*_max/min_ represent the maximum/minimum signal intensity. As shown in Fig. 1f, the dependence of g^(1)^(*τ*) matches the profile of resonant polariton signals with similar temporal distributions, indicating an intrinsic coherence transfer from the pump laser to the EPs. The buildup time (defined by the peak instant with respect to the arrival time of the pumping laser) of the coherence in EPs is found to be in the femtosecond range (∼340 fs for this specific measurement) at room temperature and the coherence duration (∼200 fs) matches with the lifetime of resonant EPs. It is the first demonstration of the coherence transfer from the driving laser field to the EPs on the femtosecond timescale.

### Buildup of non-resonant EPs and coherence transfer at high pump fluence

**Figure 2.**
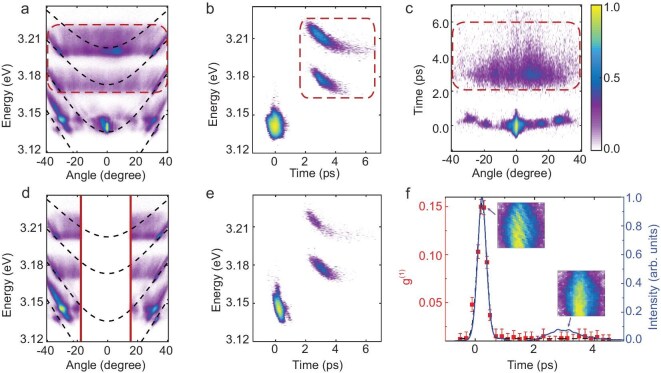
Resonant polariton excitation at high pump fluence. (a) Time-integrated angle-resolved spectra at a pump fluence of ∼5 mJ/cm^2^. (b and c) The dynamics of EPs in the energy and momentum degrees of freedom, respectively. The red dashed rectangles in (a–c) mark the signal from non-resonant EPs. (d and e) The time-integrated angle-resolved spectra and time-resolved results in the energy domain obtained at the same condition by blocking the pumping laser. (f) Time-resolved coherence and intensity of EPs. Insets show the interference patterns collected in real space after the Mach–Zehnder interferometer at time delays of ∼0 fs (left) and ∼3 ps (right).

With a higher pump fluence of ∼5 mJ/cm^2^, EPs at higher energies appear. As shown in Fig. 2a, the angular distributions for the EPs at resonant energies are wider than those obtained at the lower pump fluence. A larger initial population of the resonant EPs at the ground state of LP2 can result in a stronger stimulated scattering to other LP branches. Interestingly, EPs located at higher energies compared to the energy of the resonantly excited polaritons are observed, which are marked with a red dashed rectangle. In contrast to the narrow angular distribution obtained for the ground state condensate, the higher energy signals exhibit a distribution covering a wide angular range. The time-resolved PL in the energy and in the in-plane momentum domains are shown in Fig. 2b and c. For the EPs at resonant energy ranges, we observe a lifetime of ∼300 fs, and a scattering time of ∼450 fs at a scattering angle of 30° with an energy red shift of ∼22 meV, which indicates a stronger polariton interaction compared with those at lower pump fluence. The evolution in the energy domain can be observed more clearly by blocking the pumping laser signal, as shown in Fig. 2d and e. The high-energy signals, denoted as non-resonant polaritons (indicated by the dashed rectangles in the figures), exhibit a buildup time of about 3 ps. The lifetime of the non-resonant polaritons is much longer than those of the resonant polaritons, as shown in Fig. 2b and e, indicating different underlying processes. The features from the non-resonant part agree with the recently reported dynamics [19,20], where an excitation to the exciton reservoir and successive relaxation towards the LP branches are involved.

The measurement results for the coherence transfer are shown in Fig. 2f. In this situation, two signal peaks can be seen clearly at around 240 fs and 3 ps, corresponding to the resonant and non-resonant EPs, respectively. A clear interference pattern can be observed for the resonant peak, while coherence is lost for the later-formed non-resonant EPs. This is direct evidence that coherence of the driving laser field can be transferred to the resonantly injected polaritons, but is lost in the successive process.

**Figure 3.**
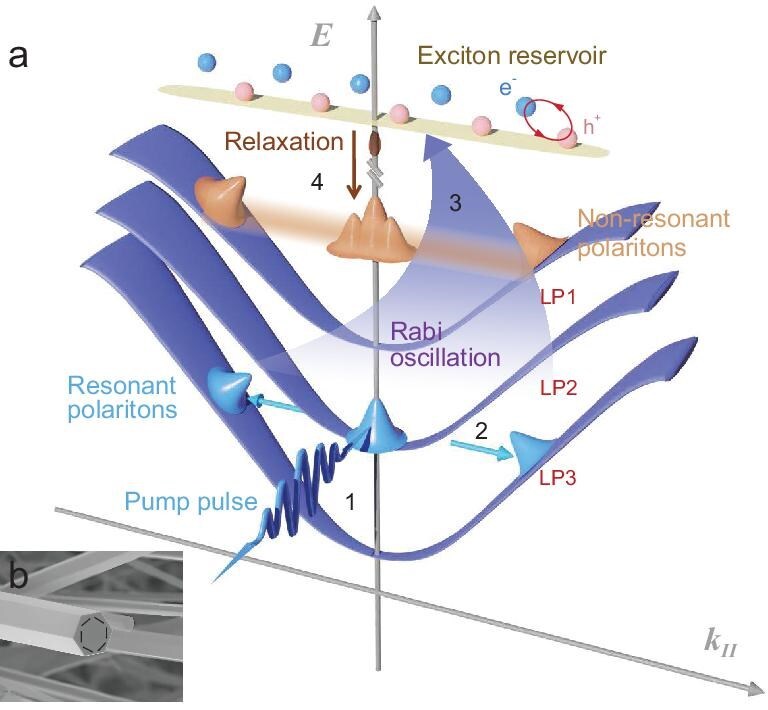
(a) A schematic diagram showing the underlying dynamics of polaritons at resonant excitation. (b) SEM image of ZnO microcavity. The black arrows represent the WGM cavity mode.

The femtosecond-resolved dynamics of the EPs following pumping are summarized in Fig. 3, which can be described as a four-step process: (i) the pulsed laser pumps a single LP branch (LP2) to inject polaritons, where resonantly injected EPs inherit the initial coherence of the pumping source; (ii) the resonant polaritons are scattered to the adjacent LPs at larger angles due to the non-linear interparticle interactions, the process of which preserves the initial coherence; (iii) once the resonant EPs are formed, the system is strongly coupled, thus the exciton reservoir will be accumulated through Rabi oscillation; and (iv) the exciton reservoir relaxes to LP branches through a decoherent stimulated emission process, realizing strong coupling with cavity photons and forming non-resonant polaritons. They appear with a wide momentum distribution as a signature of the stimulated conversion between polaritons and exciton reservoir [21]. The initial coherence of the pump source is lost in this step and not inherited by these polaritons. The experimental evidence of exciton reservoir population is included in Section II of the Supplementary data. The spatial coherence of the polariton system has been confirmed by a Michelson interferometer configuration (see Fig. S4).

To confirm this picture of the buildup and coherence transfer dynamics of the EPs, we construct a theoretical model that accounts for the two crucial processes, i.e. (i) the formation of resonant EPs and an exciton reservoir; and (ii) the formation of the high-energy non-resonant polaritons. For the formation of resonant EPs and the reservoir, a directly pumped open dissipative Gross–Pitaevskii equation is employed:


(1) \begin{eqnarray*}i\hbar {\partial }_t\left( {\begin{array}{@{}*{1}{c}@{}} {{\varphi }_c}\\ {{\varphi }_{ex}} \end{array}} \right) &=& \left[ {\begin{array}{@{}*{2}{c}@{}} {{E}_c - i{\gamma }_c}&{\Omega /2}\\ {\Omega /2}&{{E}_{ex} + g{{\left| {{\varphi }_{ex}} \right|}}^2} \end{array}} \right]\left( {\begin{array}{@{}*{1}{c}@{}} {{\varphi }_c}\\ {{\varphi }_{ex}} \end{array}} \right)\nonumber\\
&&+ \frac{{iP}}{2}\left( {\begin{array}{@{}*{1}{c}@{}} 1\\ 0 \end{array}} \right).\end{eqnarray*}

Here, *φ_c_* and *φ_ex_* are the wavefunctions of the cavity photon and exciton, respectively, ${E}_c = \frac{{{\hbar }^2{k}^2}}{{2{m}_p}} + {E}_c{|}_{k = 0}$ is the kinetic energy of the cavity photon, *E_ex_* = 3.3 eV is the exciton energy of the ZnO microcavity, *g* is the non-linear self-interaction of exciton–polaritons and *Ω* is the Rabi splitting energy between the hot exciton reservoir and the resonantly pumped cavity photons. The reservoir excitons are accumulated, accompanying the formation of the resonantly injected EPs due to Rabi oscillation. *γ*_c_ = 1 meV is the decay rate of the polaritons and *P*(*x,t*) is the pumping rate, which has a spatial and temporal dependence. The EPs at larger angles are generated predominantly through non-linear scattering from the initially induced EPs. These EP distributions have peaks at ∼20° and ∼30°, corresponding to the angle where the LP branches are populated through parametric scattering from the resonant pumping, as shown in Fig. 2a. These polariton wavepackets can inherit coherence from the pump laser, which is described by the direct coupling between *φ_c_* and *φ_ex_*, and certainly inherits the coherence from the resonant pump laser. This can be further evidenced by a measured g²(0) value of 1.18—close to 1 (see Section V in the Supplementary data), the value expected for fully coherent light.

The coherence of the pump laser, however, cannot be transferred to the later-generated non-resonant polaritons produced at higher energies. The non-resonant polaritons show up at delays of a few picoseconds and their lifetime is about one order of magnitude longer than the resonant polaritons. The reason for the coherence loss is rooted in the buildup process of these non-resonant EPs. The dynamics indicate that unlike the resonant polaritons, which are directly injected by the pumping laser, the exciton reservoir is involved in the non-resonant polariton generation. We attribute the non-resonant EP population during this resonant excitation by a decoherence model of the exciton reservoir, where the excitons relax to the LP branches and lose the initial coherence of the driving laser. The model capturing the decoherence process is given by the non-resonant Gross–Pitaevskii equations:


(2) \begin{eqnarray*}&&\nonumber\\ &&\!\!\!\!i\hbar {\partial }_t{\varphi }_{{\mathrm{LP}}0}\nonumber\\
&&\!\!\!\!= \left[\! {\begin{array}{*{20}{c}} {\displaystyle\frac{{ - {\hbar }^2{\nabla }^2}}{{2m}_0} + i\displaystyle\frac{{\hbar {R}_0{n}_{ex} - {\gamma }_c}}{2} - \displaystyle\frac{i\hbar {R}_{01}\varphi _{{\mathrm{LP}}1}^*{\varphi }_{{\mathrm{LP}}1}}{2}} \end{array}}\! \right]{\varphi }_{{\mathrm{LP}}0},\nonumber\\ \end{eqnarray*}

(3) \begin{eqnarray*}&&\nonumber\\ &&\!\!\!\!i\hbar {\partial }_t{\varphi }_{{\mathrm{LP}}1}\nonumber\\
&&\!\!\!\!= \left[\! {\begin{array}{*{20}{c}} {\displaystyle\frac{{ - {\hbar }^2{\nabla }^2}}{{2m}_1} + i\displaystyle\frac{{\hbar {R}_1{n}_{ex} - {\gamma }_c}}{2} + \displaystyle\frac{i\hbar {R}_{01}\varphi _{{\mathrm{LP}}0}^*{\varphi }_{{\mathrm{LP}}0}}{2}} \end{array}}\! \right]{\varphi }_{{\mathrm{LP}}1},\nonumber\\ \end{eqnarray*}

(4) \begin{equation*}{\partial }_t{n}_{ex} = - \left( {{\gamma }_{ex} + R_0{{\left| {{\varphi }_{{\mathrm{LP}}0}} \right|}}^2 + R_1{{\left| {{\varphi }_{{\mathrm{LP}}1}} \right|}}^2} \right){n}_{ex},\end{equation*}

where *φ*_LP_ is the wavefunction of the non-resonant polariton, m0 and m1 are the effective mass corresponding to the LP0 and LP1, respectively, *R*_0_ = 0.01 ps^−1^ μm^2^ and *R*_1_ = *R*_0_/2 are the rate of the stimulated scattering from the exciton reservoir to the LP0 and LP1 branches, *R*_01_ = *R*_0_/4 describes the bosonic cascading effect between the non-resonant polaritons formed at LP0 and LP1, and ${n}_{ex} = | {\varphi _{ex}^2} |$ is the density of the exciton reservoir, with the exciton decay rate being *γ*_ex_ = 0.2 meV. The key term is a stimulated scattering term in the form of *δ_t_φ_LP_* = *Rn_ex_φ_LP_*, where the phase information of the exciton wavefunction is absent in the conversion from the exciton reservoir to the LP polariton. Similarly, the term ${\partial }_t{n}_{ex} = R{n}_{ex}|{\varphi }_{LP}{|}^2$ indicates that the injection contains no phase information of the LP polaritons, therefore, the coherence is lost in the formation of non-resonant EPs.

**Figure 4.**
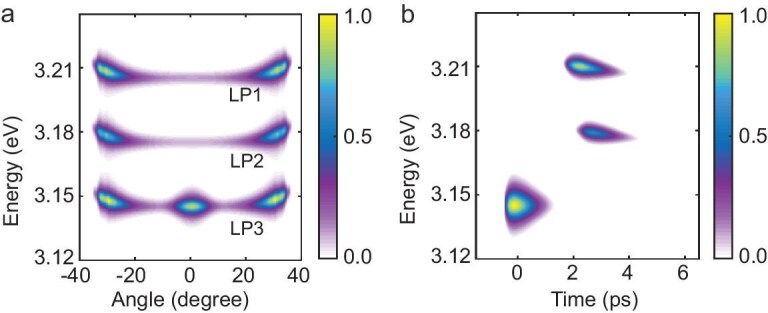
Simulation results for resonant polariton excitation at high pump fluence. (a) Calculated time-integrated angle-resolved spectra. (b) Corresponding results for time-resolved angle-integrated dynamics.

The numerical calculation results are shown in Fig. 4. The pump exhibits a Gaussian profile in both time and space with a temporal duration of 200 fs. In Fig. 4a, the time-averaged polariton emission spectrum, it can be clearly seen that the distribution agrees well with the measured result by considering the cascading effect and the parametric scattering process. The initial population of the signal and idler polaritons are generated from the acceleration of the repulsive EP interaction potential for the coherently pumped LP2 branch, as well as the formation of the non-resonantly excited LP0 and LP1 branches, strongly evidenced by the background emission signals at non-zero momenta. In Fig. 4b, the time-resolved angle-integrated polariton emission signals exhibit clear drags, as observed in the experiment, which can be explained by the decreased blueshift on account of the finite lifetime of EPs. The order of the appearance of three LP modes (first LP2, then non-resonantly transferred to LP0 and LP1), as shown in Fig. 4b, also supports the existence of the exciton reservoir decoherence process, as well as the polariton bosonic cascading effect.

## CONCLUSION

We have demonstrated the femtosecond-resolved dynamics of polariton formation with resonant pumping, and have shown unambiguous evidence of coherence transfer for resonantly injected polaritons. By exploring the dynamics of resonant excitation and successive polariton relaxation pathways, we find that the decoherence of the exciton reservoir terminates the coherence transfer and the coherence is lost in the later-produced non-resonant EPs. Time- and angle-resolved spectroscopic measurements can provide clear dynamics of the resonant polariton scattering and non-resonant polariton formation at a high pump fluence. Our work reveals the underlying mechanism of the coherence transfer in the strongly coupled light-and-matter system, and provides a solid foundation for coherence manipulation through precision control on the pumping source, which is expected to stimulate an advanced development for future polaritonic quantum devices.

## METHODS

### Material

The 1D ZnO microcavity is synthesized via a carbothermic method in a quartz furnace and growth along the c-axis, perpendicular to crystal face(0001). The diameter of microcavity is 2.9 μm and the naturally formed hexagonal cross-section provides WGMs. The large exciton binding energy (∼60 meV) of the ZnO microcavity ensures the possibility of realizing strong coupling and polariton condensation at room temperature.

### Optical spectroscopy characterizations

The detailed information is shown in Section I of the Supplementary data.

## Supplementary Material

nwaf493_Supplemental_File
