# Supplementary material for: Femtosecond coherence dynamics of exciton–polaritons
Source: Natl Sci Rev. 2025 Nov 19;13(1):nwaf493. doi: 10.1093/nsr/nwaf493 (PMC12789000; doi:10.1093/nsr/nwaf493)
Supplement: nwaf493_Supplemental_File [file nwaf493_supplemental_file.docx]

Supplemental Materials for

**Femtosecond Coherence Dynamics of Exciton Polaritons**

1. **Experimental System**


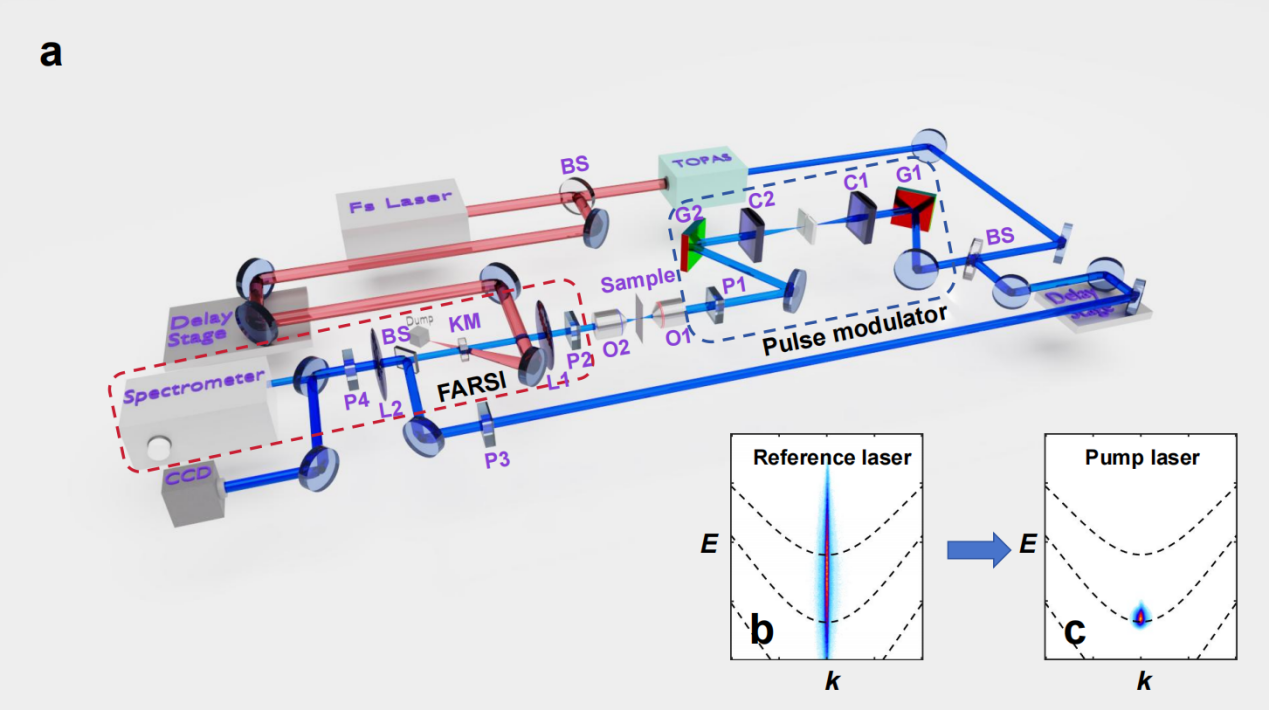


Fig. S1. (a) Schematic drawing of the experimental setup. BS represents the beam splitter. KM represents Kerr medium. G1 and G2 are gratings. C1 and C2 are cylindrical lens. O1 and O2 are objectives. L1 and L2 are lenses. P1- P4 are polarizers. (b) Spectra of the reference laser without spectral shaping. (c) Spectra of the pumping laser shaped through the 4-f pulse modulator.

Femtosecond laser pulses from a Ti: Sapphire amplification system (Coherent, Astrella, 35fs, 800nm, 1kHz) are divided into two parts. One part is sent to an optical parametric amplification system (TOPAS PRIME) to generate ultrashort pulses at the proper excitation wavelength. In our work, the central wavelength of reference laser is set at 395nm. The major part of the reference laser is sent to the 4-f pulse modulator, which can precisely shape the spectra as shown in Fig. S1(b) and (c). Afterwards, the pump laser are focused by an objective (O1, Thorlabs, LMU-15X-UVB) and are normally incident onto the ZnO microcavity. The shaped pump laser can resonantly inject a single LP branch of ZnO microcavity. The photoluminescence (PL) emission leaked from the microwire is collected by another objective (O2, Mitutoyo NUV M Plan Apo 50x, NA=0.65) and sent through a 4f system to realize angle-resolved PL spectroscopy. The signal is finally detected by an Andor spectrometer equipped with an intensified charge coupled device (iStar). Moreover, a block is placed behind O2 to block the pump laser when we measure the coherence transfer between the pumping laser and EPs. The remaining part of reference laser and the unblocked PL signals are encountered at a Charge Coupled Device(CCD) behind a Mach-Zehnder interferometer. Part of the femtosecond pulses at 800nm are guided to a Kerr medium localized along the PL pathway towards the spectrometer. Based on transient optical Kerr gating (OKG) effect, femtosecond angle-resolved spectroscopic imaging(FARSI) can be realized with a time resolution of about 50fs [18]. The dispersion of all the related optical elements in the setup have been carefully calibrated and the absolute zero time has been precisely determined [18].

1. **Evidence of exciton reservoir population under resonant excitation**

To demonstrate the formation of exciton reservoir at high energy, experimental investigations under two-pulse excitation are employed. One of the pumping pulses is tuned at about 3.14 eV (~395 nm) which is resonant with the ground state of the LP2 as the same in the main text. The other pulse is at 3.55 eV (~350 nm) which is at the non-resonant pumping energy. The intensity and relative time delay of these two pulses are precisely controlled.

For a single-pulse resonant excitation as shown in Figs. S2(a-c), there is only polariton population at more or less the same energy as the pumping pulse without non-resonant polariton population at high energy for different pumping fluences. On the other hand, a single-pulse non-resonant excitation gives the dispersion curves shown in Fig. S2(d), where the pumping fluence at 350 nm is kept below the condensation threshold. Then we turn on both pulses at a fixed time delay of about 7 ps (resonant injection first and non-resonant injection later). In these conditions a flat polariton distribution start to show up at a non-resonant higher energy above 3.2 eV in the time-integrated angle-resolved spectra (Figs. S2(e-g)) when the resonant pumping fluence exceed ~2.6 mJ/cm^2^. The fluence of the time-delayed non-resonant pumping pulse is kept below the condensation threshold.


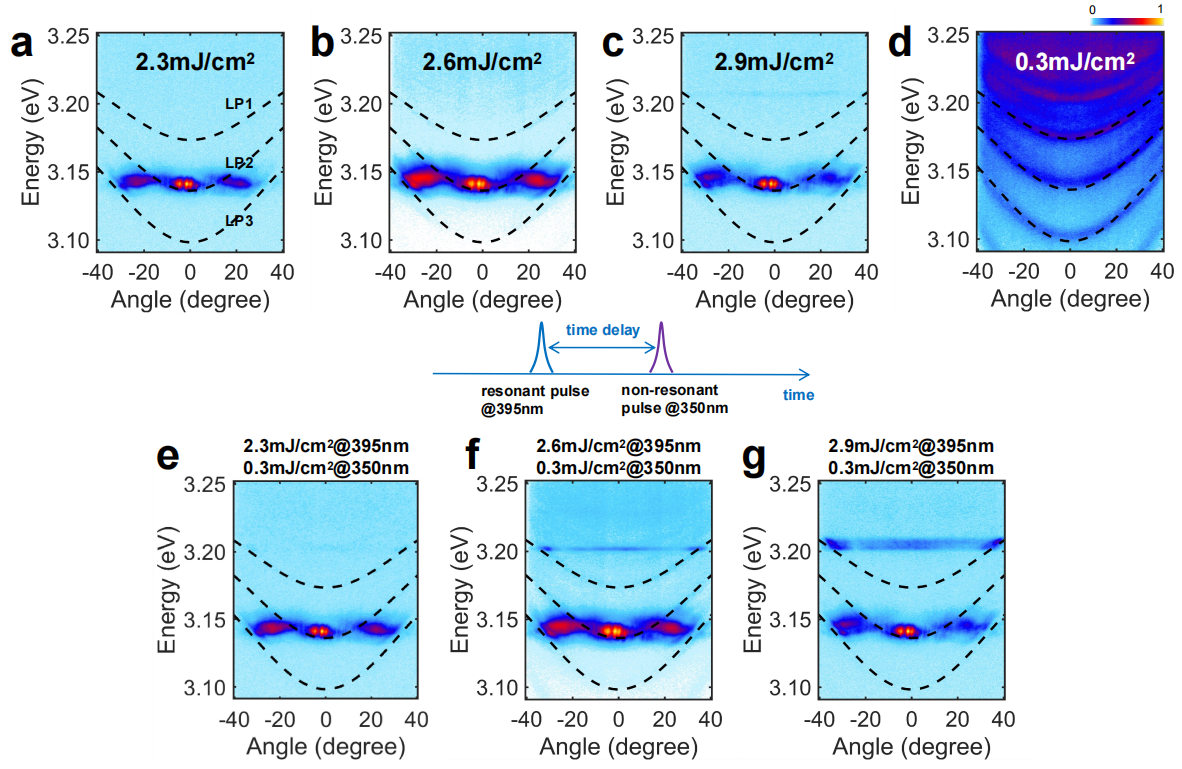


Fig. S2. Time-integrated angle-resolved spectra of exciton polaritons obtained for various experimental conditions. (a-c) Single resonant excitation at laser fluence of 2.3, 2.6 and 2.9 mJ/cm^2^ respectively. (d) The dispersion mapping obtained from single non-resonant excitation below the condensation threshold (at a fluence of about 0.3 mJ/cm^2^, ~0.4P_th_). (e-g) Bi-injection of resonant (at the same fluence in (a-c)) and non-resonant (at the same fluence in (d)) excitation at a fixed time delay of 7ps.

Since the later coming non-resonant pumping cannot produce much polariton injections by it own, but producing significant PL emission at around 3.2 eV with a resonant pre-injection. There can be an intermediate state of the exciton reservior which help to form the non-resonant polaritons. We therefore confirm the population of exciton reservoir by preforming time-resolved dynamics measurement under bi-injection. The schematic dynamic is shown in Fig. S3(a). The resonant pulse injects the ground state of LP2 and produce scattered polaritons. The Rabi oscillation process of resonant polaritons can populate the exciton reservoir. Then a weak non-resonant pulse (far below the condensation threshold) is introduced, generating hot excitons at higher energy which relax to the exciton reservoir quickly [19], serves as a supplement of exciton reservoir and stimulatedly amplifies the seed excitons produced by Rabi oscillation. Once the reservoir is supplied, the non-resonant polaritons at high energies show up, confirms an intermediate exciton reservior population from resonant injection.


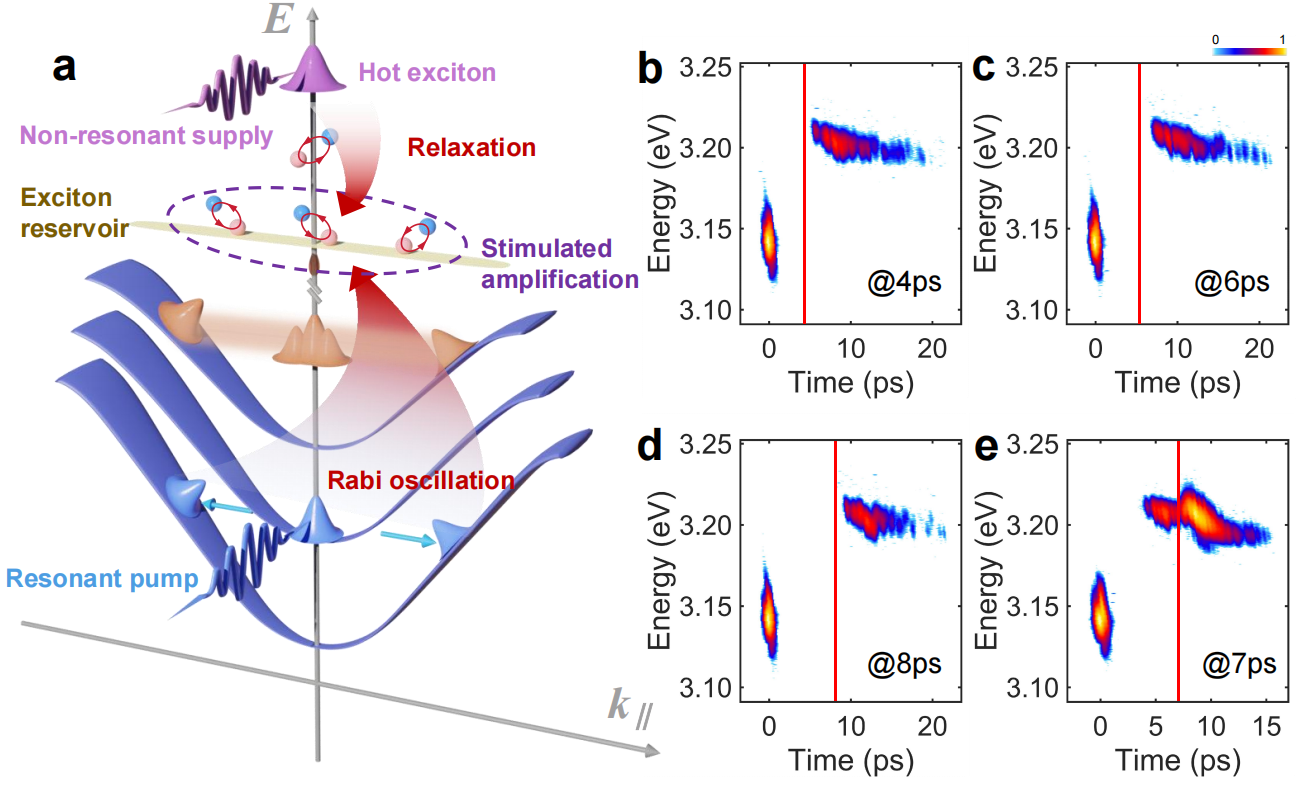


Fig. S3. (a) A schematic diagram showing the dynamics under bi-injections. The measured polariton population as a function of energy and time under bi-injection where the first resonant pumping fluence is 2.9 mJ/cm^2^ and the later arrived non-resonant pump fluence is 0.3 mJ/cm^2^ at time delays of (b) 4ps , (c) 6ps and (d) 8ps, respectively. (e) Similar to what are shown in (a-c) but at a different resonant pumping fluence ~3.2 mJ/cm^2^ and non-resonant pumping fluence ~0.58 mJ/cm^2^ at a time delay of 7 ps. The red vertical line in the figure indicates the arrival of the second pulse.

The experimental results are shown in Figs. S3(b-e). In these figures, the first resonant injection pulse at a fluence of 2.9 mJ/cm^2^ arrives at 0 time delay, while the arrival time of the second pulse at non-resonant energy is varied and indicated by the red vertical line. The resonant injection itself is not sufficiently strong to produce polaritons at higher energy (~3.2 eV). However, with the successive non-resonant injection at very low laser fluence, polaritons at 3.2 eV can be populated. We can see that the appear time of the non-resonant polaritons at around 3.2 eV is strongly dependent on the arrival time of non-resonant pumping as shown in Figs. S3(b-d). However, the birth time of the high energy polaritons are delayed by ~1ps with respect to the arrival time of the second injection pulse, which indicates the existence of the exciton reservoir. Due to the fact that the non-resonant pumping itself cannot produce polariton BEC (shown in Fig. S2(d)), but a stimulated amplification can take place with the pre-population of the exciton reservoir by the early arrived resonant pumping pulse. The amplified reservoir can then inject certain LP branches to produce polaritons which can be observed. This build-up process of polaritons from the exciton reservoir takes picoseconds, which agree with the measured dynamics. This could be an indirect evidence that the exciton reservoir is populated by the resonant excitation first, when supplied by non-resonant injection at a proper delay, extra non-resonant polaritons can be produced through stimulated amplification of the exciton reservoir.

For comparison, we run the time-resolved measurements at a stronger resonant injection where polaritons at higher energy can be produced by the first resonant injection itself. Then we can see that the second weak non-resonant injection(well below the condensation threshold) can cause an instantaneous enhancement at the polariton population as soon as it arrives. A clear blueshift can be recognized for the enhancement as shown in Fig. S3(e). The amplification of non-resonant polaritons usually occur very quickly. The time delay show distinct features whether the exciton reservoir or the polariton is amplified by the successive injection.

1. **Spatial coherence of the polaritons**


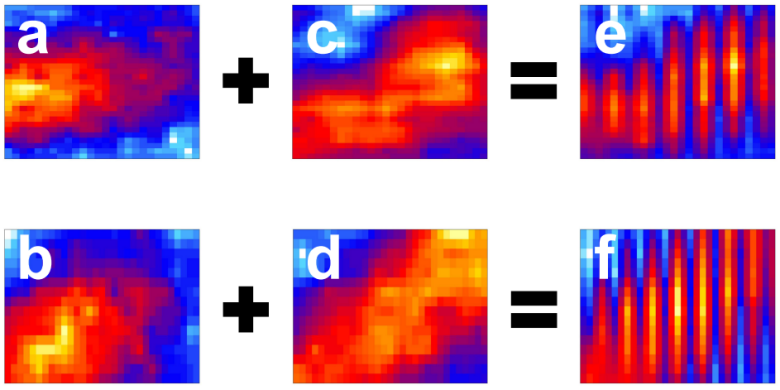


Fig. S4. Interferograms recorded in a Michelson interferometer in the retro reflector configuration. (a,b) Real space PL image obtained from one arm of Michelson interferometer in the same configuration of Fig. 1(d) and Fig. 2(d). (c,d) Real space PL image from the second arm by using a retroreflector to flip the image of (a) and (b) in a centro-symmetric way. (e,f) Interference pattern after superposition of the images from the two arms of the Michelson interferometer.

The buildup of spatial coherence lays the heart of polariton system, and is visualized through a Michelson interferometer. The PL emission of polaritons under different pumping powers are sent to the Michelson interferometer with one of the arms replaced by a retroreflector to invert the image centero-symmetrically. The use of retroreflector enables the measurement of g^(1)^(r, -r) extract from the interference fringe contrast between points r and -r from the spot centre. Fig. S4(a) and (c) is the real space PL image of Fig. 1(d) and its inverted image, respectively. Clear fringes are observed in the superposition of two arms (shown in Fig. S4(e)), indicating the buildup of spatial coherence of resonant polaritons. For the mixture of non-resonant and resonant polaritons in Fig. 2(d), the clear interferences preserved as shown in Fig. S4(f), demonstrating the buildup of spatial coherence in the system.

1. **The spatial coherence between EPs at large degrees**

To confirm the coherence between the signals at large angles, we carried out Mach-Zehnder interferometric measurements for the signals at *~*±30 degrees and the results are shown in Fig. S5. The interference fringes for two distinct energy ranges, i.e. around 3.15 eV and 3.21 eV, were obtained and presented in Figs. S5(d) and S5(g), indicating the signals at ~±30 degrees are coherent. The interference fringes shows up for both the resonant parts (labeled by 3,4) and the non-resonant parts (labeled by 1,2) The coherence time for this two parts were extracted from the temporal width of the fringe contrast. As shown in Fig. S6, they are about 0.94 ps and 1.11ps for the non-resonant and the resonant parts, respectively. The confirmation of spatial coherence supports the contribution of parametric scattering process and potential acceleration, both of these processes exhibit macroscopic coherence [22, 23].


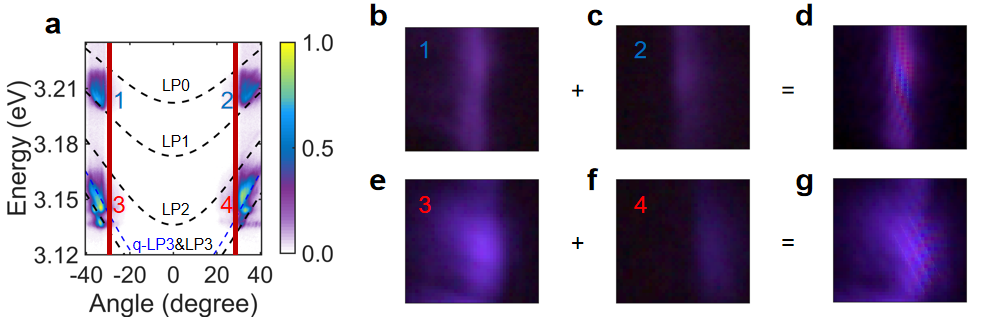


Fig. S5 The interference fringes for EPs at positive and negative angle ranges. (a) The steady-state angle-resolved spectra with a blocking region of about -30° to 30°. 1/3 marks the non-resonant/resonant EPs at around -30° to -40°. 2/4 marks the non-resonant/resonant EPs at around 30° to 40°. The black and blue dashed parabolas mark the polariton WGM(LP0,1,2,3) and quasi-WGM LP(q-LP3) branches. (b, e) The real space PL emissions from non-resonant and resonant EPs at around -30° to -40°, respectively. (c, f) The real space PL emissions from non-resonant and resonant EPs at around 30° to 40°, respectively. (d) The interference fringes collected by overlapping two arms (b, c) in a Mach-Zehnder interferometer at zero time delay. (g) The interference fringes collected by overlapping two arms (e, f) in a Mach-Zehnder interferometer at zero time delay. The gray dashed lines mark the interference fringes.


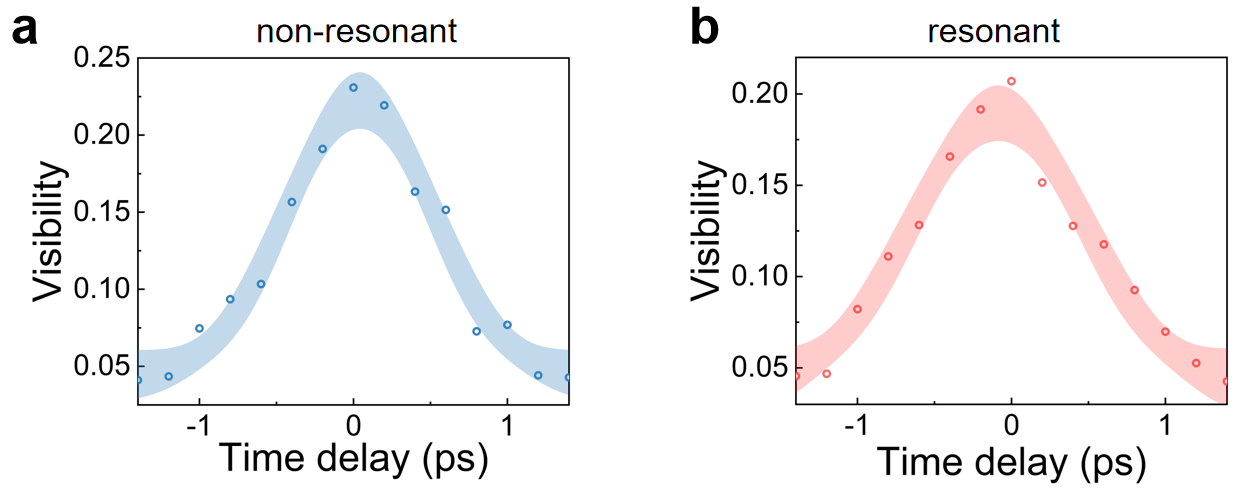


Fig. S6 The time-dependence of the fringe visibility for selected EPs in (a) the non-resonant and (b) the resonant ranges. The experiment data were fitted with a Gaussian function with 95% CI.

1. **g^(2)^(0) measurement for resonant and non-resonant EPs**

To fully characterize the nature of polariton populations, we carried out the measurement of g^(2)^(0) factor, which can be obtained by inserting our time-resolved data I(t) into the function g^(2)^(τ)=<I(t+τ)I(t)>/[<I(t+τ)><I(t)>], where I(t) is the polariton emission intensity at time t, and <...> indicates the time averaging. To avoid the interference of pump laser, we select resonant EPs at large angles (marked by red dashed circles) in Fig. S7(a) to derive the g^(2)^(0) value. The g^(2)^(0) turns out to be 1.18 for the resonant EPs, indicating these EPs are coherent. For the non-resonant EPs, as marked by the black dashed rectangle in Fig. S7(a), the g^(2)^(0) is about 2.44, showing a superbunching feature. These non-resonant signals contain EPs from multiple LP branches. The corresponding energy resolved dynamics are shown in Fig. S7(b). The g^(2)^(0) for P1 and P2 LP branches are derived to be about 2.25 and 3.01, respectively. The g^(2)^(0) factors for both the non-resonant LP branches are larger than 2, indicating the existence of bosonic cascading effect for non-resonant EPs.


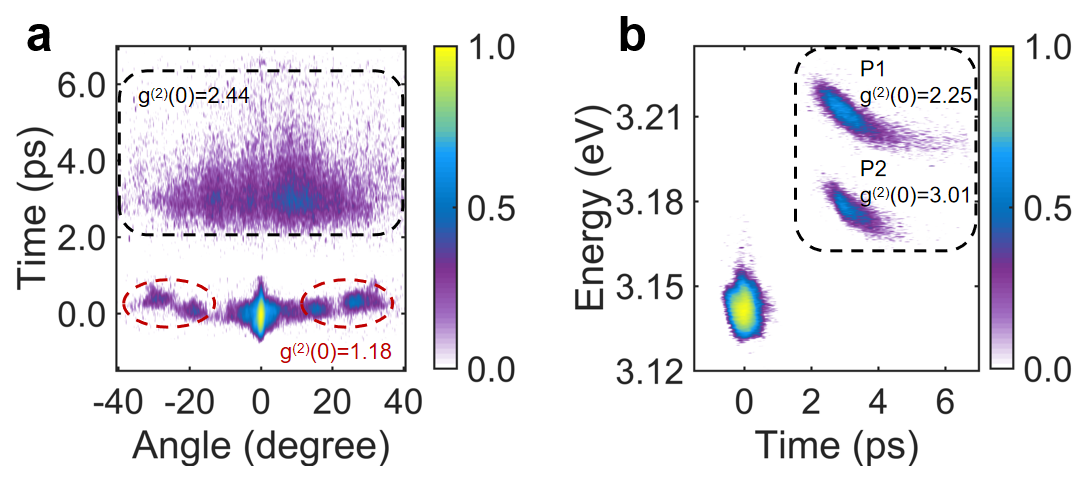


Fig. S7 The g^(2)^(0) factors for the resonant and non-resonant EPs. (a) The angle-resolved dynamics at high pump fluence. The red dashed circle marks the selected resonant EPs utilized in g^(2)^(0) measurement. The black dashed rectangle marks the non-resonant EPs in g^(2)^(0) measurement. (b) The energy-resolved dynamics at high pump fluence. The g^(2)^(0) factors of each branch(labeled P1 and P2) of non-resonant EPs were measured.

1. **Theoretical modeling**

(A). Theoretical description of the strong light-matter coupling system

The key feature of the 4-step process can be well picturally descripted by the coupled oscillator model of exciton-polariton system:

$$H=\left[ \begin{matrix} E_{c0} & 0 & 0 & 0 & 0 & \Omega/2 \\ 0 & E_{c1} & 0 & 0 & 0 & \Omega/2 \\ 0 & 0 & E_{c2} & 0 & 0 & \Omega/2 \\ 0 & 0 & 0 & E_{c3} & 0 & \Omega/2 \\ 0 & 0 & 0 & 0 & E_{c4} & \Omega/2 \\ \Omega/2 & \Omega/2 & \Omega/2 & \Omega/2 & \Omega/2 & E_{ex} \end{matrix} \right]$$

where $E_{ci}=\frac{\hbar^{2}k^{2}}{2m_{i}}+E_{ci}|_{k=0} (i=0,1,2,3,4)$, is the dispersion of cavity photon, $E_{ex}=3.3 \mathrm{eV}$ is the exciton energy of ZnO, $\Omega=300 \mathrm{meV}$ is the Rabi splitting energy between exciton reservoir and cavity photons in ZnO nanowires. $E_{ci}|_{k=0}$ is the characteristic energy of the quasi-1D hexagonal ZnO WGM cavity, which can be calculated from the formula

$$E_{ci}|_{k=0}=\frac{hc}{3\sqrt{3}nR}\left[ N+\frac{6}{\pi}\arctan\left( n^{-1}\sqrt{3n^{2}-4} \right) \right],$$

where $R=3\mathrm{um}$ is the sidelength of hexagonal ZnO microcavity, $n=2.24$ is the refractive index at 400 nm wavelength, $N$is the number of the cavity modes corresponding to $E_{c0-4}$. The energy difference between two neighboring cavity modes is $\Delta E=\frac{hc}{3\sqrt{3}nR}=35\mathrm{meV}$, which leads to a ~35 meV gap between two neighboring polariton modes (see Fig. S8).


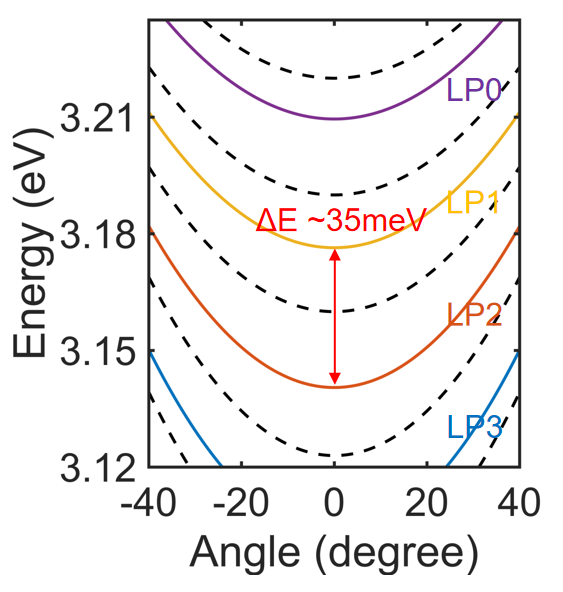


Fig. S8. Polariton dispersions calculated from the coupled oscillator model. Dashed black curves represent cavity photon dispersions, while the solid curves are polariton dispersions after diagonalizing the Hamiltonian matrix. The energy gap between two nearest polariton modes is about 35 meV.

The buildup dynamics of the resonant and non-resonant exciton-polaritons can be described within the framework of the coupled oscillator model by introducing the pump $F$ and decay $\gamma$. Notice that the resonant pumping only creates cavity photons at $E_{c2}$, therefore the basis for the resonant pumping is $\left( \begin{matrix} 0 & 0 & 1 & 0 \end{matrix} \begin{matrix} 0 & 0 \end{matrix} \right)^{T}.$ The evolution equation is

$$i\hbar\partial_{t}\Psi=H\Psi+iF\cdot\left( \begin{matrix} 0 \\ 0 \\ \begin{aligned} 1 \\ 0 \\ 0 \\ 0 \end{aligned} \end{matrix} \right)-i\gamma\Psi,$$

where the pulse pump has a half-width 100 fs in time. The result of the population for exciton and cavity photons is shown in Fig. S9. It can be seen that on account of the Rabi splitting arising from the photon-exciton strong coupling, the resonant pumping at $E_{c2}$ can be effectively transferred to population of other possible polariton modes and excitons. Besides, with the increase of the pump power, the population of polaritons becomes higher, which qualitatively explains the transfer dynamics between resonant and non-resonant polaritons through the exciton reservoir.


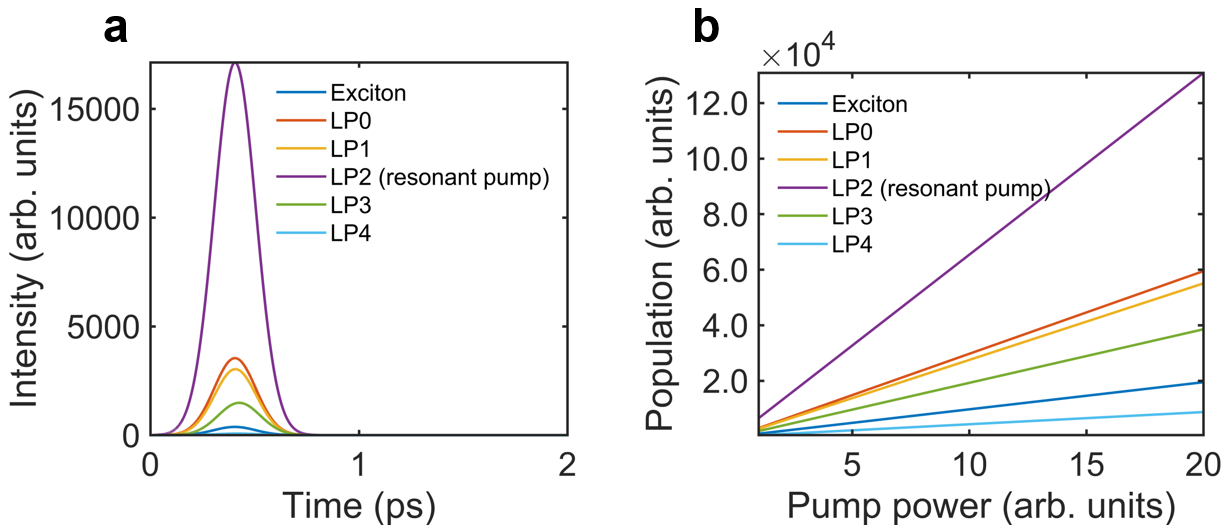


Fig. S9. Exciton and cavity photon populations. (a) Populations evolution after injection of one pulse pump. (b) Pump power dependence, with the unit normalized.

(B). Gross-Pitaevskii equation for resonant and non-resonant pumping

As is shown in the main text, the GPE composed of the resonant polariton wavefunction, exciton density and non-resonant polariton wavefunction due to the decoherence and cascading writes as equation (1-4). The pump power dependence can be seen that due to the strong light-matter coupling, the population of the polariton grows as the pump power increases. Due to the nature of the resonant excitation, there is no “pump threshold” for the resonant polariton, as the driving term leads to an exponential increase of the resonant polariton density. With the increase of the density of the exciton reservoir, the stimulated scattering between exciton reservoir and non-resonant polaritons becomes significant (decoherence process). Above the threshold (where $P_{\mathrm{th}}\approx\frac{\gamma_{c}\gamma_{ex}}{R}\approx{\gamma_{ex}n}_{ex}$), one can observe the signal from the non-resonant polaritons (see Fig. S10). This also explains the disappearance of the non-resonant polariton signal in the low pump power regime.


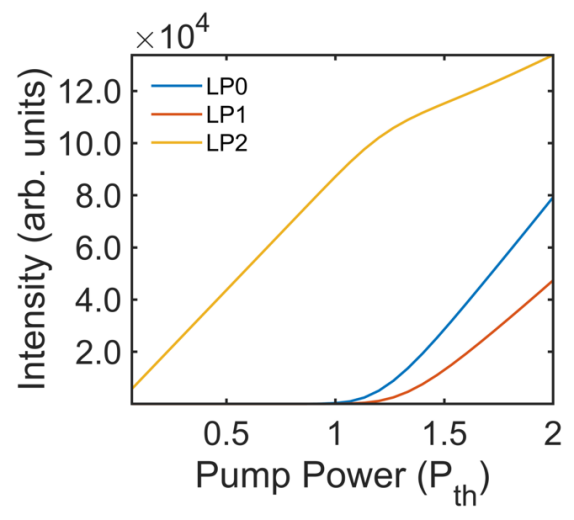


Fig. S10. Pump power dependence of three LP modes. The power threshold here represents the threshold of the non-resonant EPs population.

(C) The non-resonant polariton dispersions

As is discussed above, the non-resonant polaritons gain from the stimulated emission of the exciton reservoir, in which process they loss the coherence. The two main signatures of the non-resonant polariton dispersions are

(1) non-zero background signal;

(2) EP population at certain angles.

The signature (1) is the result of the stimulated conversion between the exciton reservoir and the non-resonant pump, which was already discussed by Wouters et. al[21]. The exciton reservoir will maintain some geometric characters of the of the pumping. Under the effect of the EP self-interaction, polaritons are scattered towards large momentum states, featured with a wide background of emission signals. Meanwhile, this process grants the initial populations at large angles, which amplifies the parametric scattering process in the 1D ZnO microcavity, even without the probe pulse. The parametric scattering further increases the population at certain angles through the repulsive polariton-polariton interaction, leading to signature (2).

(D) The parametric process in resonant and non-resonant EPs

The optical parametric oscillation arisng from the second-order non-linearity of the polariton self-interaction is taken into consideration in the reciprocal space of the polariton wavefunction:

$$i\hbar\partial_{t}\hat{\varphi}_{\mathrm{LP}i}\left( 0 \right)={2E}_{\mathrm{OPO}}\hat{\varphi}_{\mathrm{LP}i}^{*}\left( 0 \right)\hat{\varphi}_{\mathrm{LP}i+1}\left( -k_{i+1} \right)\hat{\varphi}_{\mathrm{LP}i+1}\left( k_{i+1} \right)$$

$$i\hbar\partial_{t}\hat{\varphi}_{\mathrm{LP}i+1}\left( {\pm k}_{i+1} \right)=E_{\mathrm{OPO}}\hat{\varphi}_{\mathrm{LP}i}^{2}\left( 0 \right)\hat{\varphi}_{\mathrm{LP}i+1}^{*}\left( \mp k_{i+1} \right)$$

where $E_{\mathrm{OPO}}=1 \mathrm{meV}\mathrm{um}^{-2}$ is the energy of the optical parametric oscillation, $i=\{0,1,2\}$ is the index of two non-resonant polariton branch and one resonant polariton branch, $\hat{\varphi}=\int e^{ikx}\varphi(x)dx$ is the wavefunction in reciprocal space, ${\pm k}_{i}$ are the wavevectors of the final scattered signal and idler states. In order to solve the system of Gross-Pitaveskii equations, time splitting spectral method is applied for the numerical calculation with the kinetic Laplacian operator and the parametric scattering solved in the reciprocal space.
